# Impact of a brief series of soccer matches on vascular conditions in youth women

**DOI:** 10.3389/fphys.2024.1476627

**Published:** 2024-11-27

**Authors:** Jun Sugawara, Nana Ogoh, Hironori Watanabe, Shotaro Saito, Maki Ohsuga, Tetsuya Hasegawa, Narumi Kunimatsu, Shigehiko Ogoh

**Affiliations:** ^1^ Human Informatics and Interaction Research Institute, National Institute of Advanced Industrial Science and Technology, Tsukuba, Japan; ^2^ Department of Applied Nutrition, Kagawa Nutritional University, Saitama, Japan; ^3^ Chifure AS Elfen Saitama, Saitama, Japan; ^4^ Department of Biomedical Engineering, Toyo University, Kawagoe, Japan

**Keywords:** finger photoplethysmogram, arterial stiffness, wave reflection, soccer, women, repeated-measures correlation analysis

## Abstract

**Background:**

Accumulative excessive physical load elevates central arterial stiffness and smooth muscular tone of peripheral vascular beds in endurance athletes. The aim of this study was to test the hypothesis that a brief series of soccer matches would increase central arterial stiffness and arterial wave reflection from the periphery in young female football players.

**Methods:**

Fifteen subjects (17.2 ± 0.7 years, mean ± SD) participated in four matches over five consecutive days (one match per day, with two consecutive days of matches followed by one rest day, repeated twice) in the Youth Girls Soccer Tournament, either as starters or substitutes. Heart rate, blood pressure (BP), and the second derivative of the photoplethysmogram (SDPTG) were assessed the night before and 4 h after each match. The ratios of the first and second descending waves to the first ascending wave of SDPTG (B/A ratio and D/A ratio) were calculated as indices of central arterial stiffness and peripheral wave reflection, respectively. The intra-individual relationship among interest variables was evaluated using the repeated-measures correlation analysis (rmcorr).

**Results:**

Post-match D/A ratio, systolic and diastolic BP were lower compared to the pre-match value, while the B/A ratio did not change significantly. Heart rate was higher post-than pre-match. Rmcorr demonstrated significant intra-individual correlations of the D/A ratio with diastolic BP (r_rm_ = 0.259, P = 0.008) and heart rate (r_rm_ = −0.380, P< 0.001).

**Conclusion:**

Contrary to our hypothesis, a brief series of matches did not increase central arterial stiffness in young female football players. Instead, the matches induced a repeated, temporary attenuation of arterial wave reflection. This attenuated arterial wave reflection from the periphery appeared to be associated with reduced diastolic BP and a compensatory increase in heart rate.

## 1 Introduction

Accumulative excessive physical load increases central arterial stiffness and smooth muscular tone in the peripheral vascular beds of endurance athletes, likely due to enhanced sympathetic nervous activity and oxidative stress (Iellamo et al., 2002; Knez et al., 2006; Barnes et al., 2010; Vlachopoulos et al., 2010). We previously reported that, in well-trained male collegiate endurance runners, aortic systolic and pulse pressures, along with central arterial stiffness, increased after a 7-day intense endurance training camp, with these changes correlating to the total running distance (Tomoto et al., 2015; 2018). Therefore, the vascular condition—characterized by central arterial stiffness and peripheral vascular smooth muscle tone—may reflect exercise-induced physiological fatigue and recovery.

It should be noted that postexercise hemodynamic responses to acute endurance exercise bouts differ between men and women (Senitko et al., 2002). However, our studies subjected male-only groups, and thus, the hemodynamic response to repeated, intense endurance exercise bouts in women is not entirely known.

A photoplethysmogram (PTG) measures changes in the absorbance of hemoglobin based on the Lambert–Bernstein law, indicating regional blood flow (Jespersen and Pedersen, 1986). The waveform, recorded at the finger, has two main components: an early systolic wave caused by left ventricular ejection, and a reflected wave from the periphery (Westerhof et al., 1972a; Hashimoto et al., 2002). These components could be characterized by the five inflection points on PTG waves using second derivatives (e.g., SDPTG). This technique provides a composite measure of vascular aging (Takazawa et al., 1998), and the detailed wave analysis offers surrogate markers for both central arterial stiffness and peripheral vascular smooth muscle tone (Westerhof et al., 1972b; Hashimoto et al., 2002). Thus, it is a valuable tool for assessing the vascular condition of athletes.

Athletes competing at higher levels require intense, high-volume training, which often results in prolonged imbalances between fatigue and recovery. This can reduce trainability, impair performance, and increase the risk of overtraining syndrome (Lee et al., 2017). Ideally, the relationship between training load and physiological responses should be monitored individually. However, much of the current evidence is based on cross-sectional studies, which fail to capture intra-individual changes over time (Lee et al., 2017). Additionally, conventional correlation analysis applied to repeated measures can produce biased results due to non-independent observations (Bakdash and Marusich, 2017).

In this field study, we sought to identify sensitive vascular biomarkers that reflect physical fatigue and recovery in athletes. We aimed to elucidate the impact of a brief series of matches on vascular conditions in young female soccer players. Additionally, we determined the relationship between intra-individual responses of vascular condition, hemodynamics, and fatigue to the repetitive matches using the repeated measures correlation analysis (Bakdash and Marusich, 2017). Given that the required running speed and distance in soccer vary depending on a player’s position and the strength of the opposing team, this approach allowed us to efficiently clarify the individual relationship between physical fatigue and vascular responses. We hypothesized that a brief series of soccer matches would increase central arterial stiffness and arterial wave reflection from the periphery in young female football players, correlating with fatigue, and that this vascular condition would be associated with hemodynamic markers such as blood pressure and heart rate.

## 2 Methods

### 2.1 Participants

Fifteen female football players (mean age: 17.2 ± 0.7 years) participating in the Youth Girls Soccer Tournament (XF CUP 2021 Japan Club Youth U18) underwent evaluation. None of the participants had cardiovascular, cerebrovascular, and respiratory disease. All participants were not taking any prescribed or over-the-counter medications or supplements. All procedures conformed to the Declaration of Helsinki (Seventh revision, 64th Meeting, Fortaleza, 2013) and were approved by the Institutional Review Board at Toyo University (Approval Number: TU 2021-022). Th e participants (or parents/guardians) provided written informed consent before participation.

### 2.2 Experimental procedure

**FIGURE 1:**
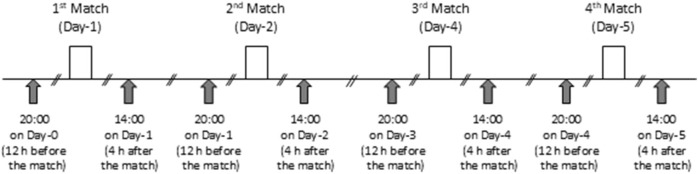
Experimental timeline.

All subjects participated in four matches over five consecutive days (one match per day, with two consecutive days of matches followed by one rest day, repeated twice) in the Youth Girls Soccer Tournament, either as starters or substitutes. Throughout the tournament period, the following measurements were performed before night and approximately 4 h after each match (Figure 1). These measurement timings correspond to 2 h after dinner and 2 h after lunch. Body weight and fat (via a digital scale for body weight, TANITA, Japan), single isometric knee extension strength (KES, via a goniometer, mobie MT-100 and -250; Sakai Medical, Tokyo, Japan), and the rate of perceived exertion (RPE) were also measured. RPE was evaluated by the visual analog scale. Each participant marked their subjective whole body fatigue level on a 100 mm black line (the left end is “0” and the right end is “100”).

### 2.3 Vascular measurements

**FIGURE 2:**
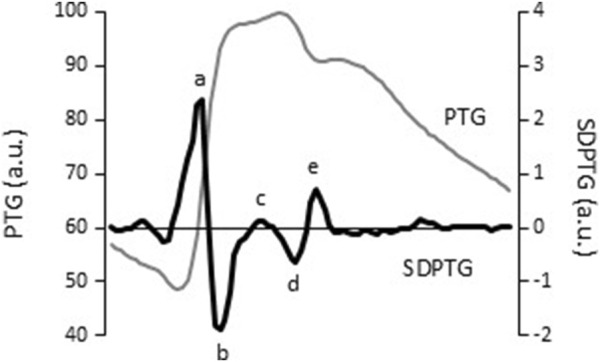
Sample signals of finger photoplethysmogram (PTG, grey line) and the second derivative of the finger photoplethysmogram (SDPTG, black line). Letters of “a” - “e” indicate five inflection points on finger PTG waves characterized by the SDPTG: the initial positive (a), early negative (b), re-increasing (c), late re-decreasing (d), and diastolic positive waves (e).

After 5 min of quiet sitting on the chair, hemodynamic variables were collected in the same posture. All participants underwent heart rate (Bedside monitor BMS-3400; Nihon Kohden, Tokyo, Japan), blood pressure (Intellisense, Omron Healthcare, Kyoto, Japan), and PTG recordings. Stable pulse waveforms were stored for 30 s on the right index finger with a customized fingertip Photoplethysmography (Alps, Tokyo, Japan) and calculated the second Derivative waveform of PTG (i.e., SDPTG) using an optimized software (AGVS monitor, Alps, Tokyo, Japan). The methodology details for measuring PTG and SDPTG are reported (Takazawa et al., 1998; Fukuie et al., 2021). The SDPTG provides 5 specific inflection points on PTG waves such as the initial positive wave (“a” wave), an early negative wave (“b” wave), a re-increasing wave (“c” wave), a late re-decreasing wave (“d” wave), and a diastolic positive wave (“e” wave) (Figure 2). The amplitude ratio of the early negative wave (“b” wave) to the initial positive wave (“a” wave) (i.e., B/A ratio) is assumed as the index of central arterial stiffness, whereas that of the late re-decreasing wave (“d” wave) to the “a” wave (i.e., D/A ratio) is closely related to the intensity of wave reflection from the periphery, and thus, peripheral vascular tone (Westerhof et al., 1972b; Hashimoto et al., 2002). The validation of this automatic device and its reproducibility have been reported previously, with an excellent intra-observer reproducibility evaluated by intraclass correlation (B/A ratio = 0.868, D/A ratio = 0.793, vascular age = 0.914) (Fukuie et al., 2021).

### 2.4 Statistical analysis

All the analyses were conducted with IBM SPSS Statistics (ver. 29.0, IBM Corp., Chicago, IL) and RStudio (2023.09.1 Build 494, Posit Software, PBC). The P-values <0.05 were considered to denote statistical significance. The linear mixed model (LMM), which can appropriately handle cases where some data is missing within repeated measures, was used to analyze the main and interaction effects of the match (the first to fourth matches) and time (pre-vs post-match) on body weight, hemodynamic variables, KES, and RPE. Post hoc analysis using Bonferroni corrections was applied when main factors or interactions were significant. Data are expressed as estimated marginal means and 95% confidential intervals. The intra-individual relationship among interest variables was evaluated using the repeated-measures correlation analysis (rmcorr) using the R package (Bakdash and Marusich, 2017).

## 3 Results

During the experimental period, there were a total of two instances of missing data for both B/A and D/A ratios. Additionally, there were four instances of missing data for RPE and seven instances for KES. Therefore, estimated marginal means and 95% confidence intervals were reported using LMM. As shown in Table 1, body weight was lower (time effect: P< 0.0001), and RPE was higher (time effect: *p* = 0.001) post-match compared with pre-match. KES tended to be lower post-match than pre-match (time effect: *p* = 0.084).

**TABLE 1:** Responses of physiological indices to the repetitive soccer matches.

	Match	Pre-match		Post-match		P-value for	
		EMM	95% CI	EMM	95% CI	Linear Mix Model	
Body weight	1st	53.5	(51.2–55.8)	52.7^b^	(50.4–55.0)	Match	**0.007**
kg	2nd	53.1	(50.8–55.4)	52.7^b^	(50.4–55.0)	Time	**<0.0001**
	3rd^a^	52.7	(50.4–55.0)	52.6	(50.3–54.9)	Interaction	**0.017**
	4th	53.4	(51.1–55.7)	52.8^b^	(50.5–55.1)		
KES	1st	29.8	(24.9–34.7)	27.8	(23.1–32.5)	Match	0.942
kg	2nd	30.0	(26.7–33.4)	26.4	(21.7–31.2)	Time	0.084
	3rd	28.4	(23.6–33.2)	28.6	(23.9–33.3)	Interaction	0.384
	4th	28.9	(24.1–33.7)	27.5	(22.8–32.3)		
RPE	1st	46.6	(38.1–33.7)	60.5	(52.1–68.9)	Match	0.017
a.u.	2nd	54.5	(46.1–62.9)	71.9	(63.6–80.1)	Time	**0.001**
	3rd	51.2	(42.9–59.4)	68.5	(60.3–76.8)	Interaction	0.158
	4th	59.5	(51.3–67.8)	67.8	(59.6–76.1)		
Heart rate	1st	68	(63–73)	73	(68–78)	Match	0.725
bpm	2nd	65	(61–70)	73	(68–78)	Time	**0.019**
	3rd	68	(63–73)	73	(68–78)	Interaction	0.476
	4th	68	(63–73)	70	(65–75)		
Systolic BP	1st	111	(105–116)	103	(97–109)	Match	0.700
mmHg	2nd	106	(100–112)	105	(99–110)	Time	**0.021**
	3rd	107	(101–113)	106	(100–111)	Interaction	0.267
	4th	106	(100–112)	102	(96–108)		
Diastolic BP	1st	67	(62–72)	58	(53–63)	Match	0.854
mmHg	2nd	61	(56–66)	61	(56–66)	Time	**0.016**
	3rd	62	(57–67)	61	(56–66)	Interaction	0.099
	4th	64	(59–69)	60	(55–65)		
B/A ratio	1st	0.73	(0.66–0.79)	0.76	(0.70–0.83)	Match	0.548
a.u.	2nd	0.82	(0.76–0.88)	0.77	(0.71–0.83)	Time	0.653
	3rd	0.78	(0.71–0.84)	0.77	(0.71–0.83)	Interaction	0.383
	4th	0.77	(0.71–0.83)	0.75	(0.69–0.82)		
D/A ratio	1st	0.25	(0.19–0.32)	0.19	(0.12–0.25)	Match	**0.009**
a.u.	2nd	0.23	(0.16–0.29)	0.15	(0.08–0.22)	Time	**0.023**
	3rd^a^	0.14	(0.08–0.21)	0.14	(0.07–0.20)	Interaction	0.134
	4th^a^	0.19	(0.13–0.26)	0.10	(0.03–0.17)		

EMM, estimated marginal mean; CI, confidential interval; KES, single isometric knee extension strength; BP, blood pressure.

^a^ Significant difference vs. 1st match.

^b^ Significant difference vs. pre-match.

**FIGURE 3:**
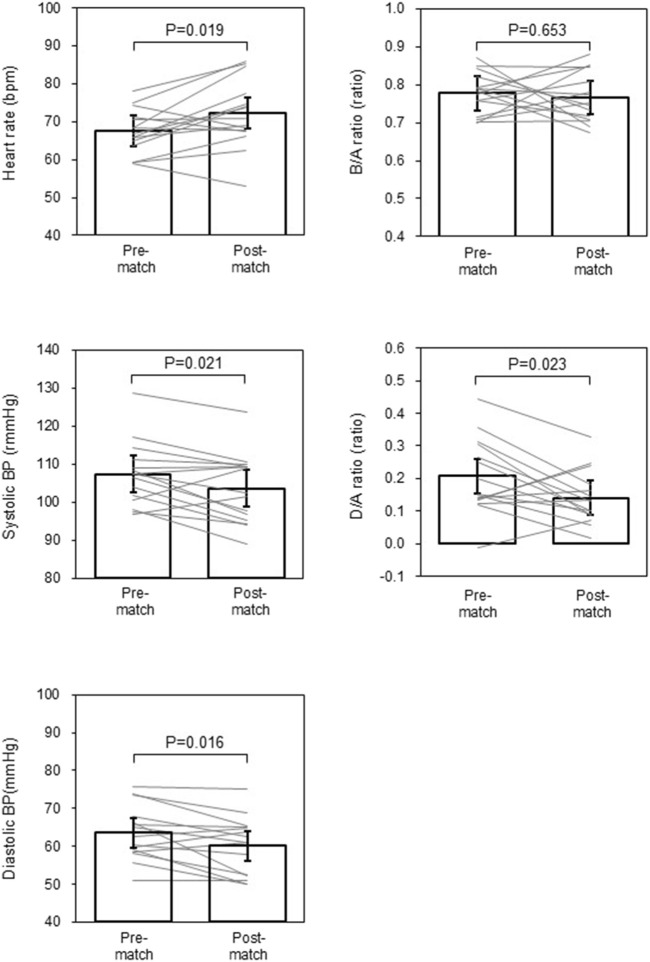
Hemodynamic variables and the second derivative of the finger photoplethysmogram indices pre- and post-match. Data are estimated marginal means and 95% of confidential intervals. Grey lines indicate changes in individual estimated marginal mean.

**FIGURE 4:**
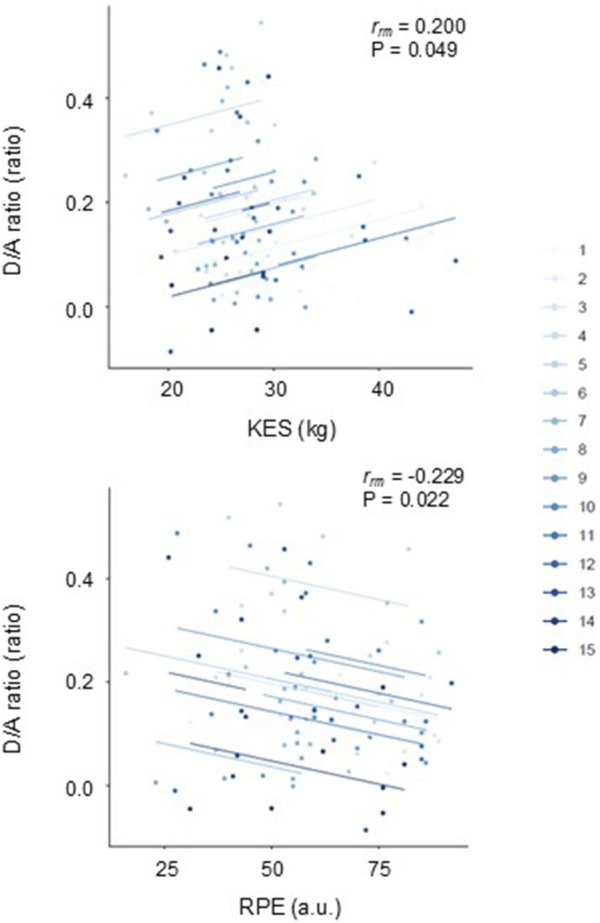
Repeated measures correlation plots of the single isometric knee extension strength (KES) (*top panel*) and the rate of perceived exertion (RPE) (*bottom panel*) with the D/A ratio.

**FIGURE 5:**
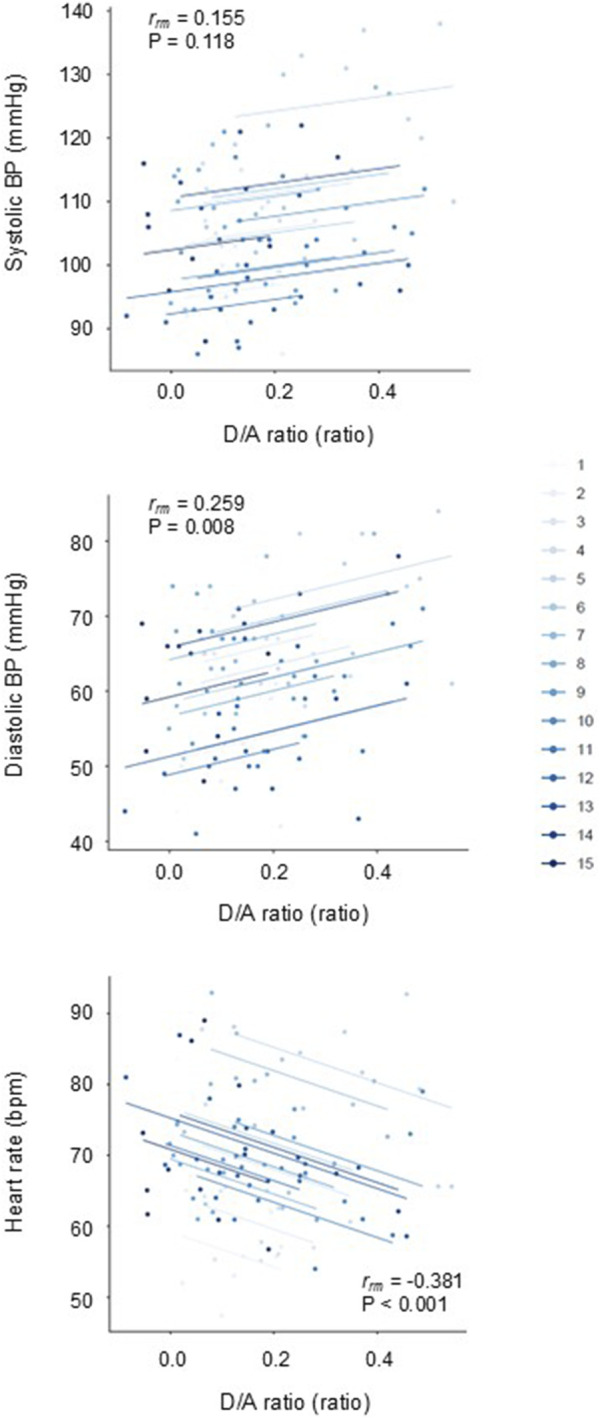
Repeated measures correlation plots of the D/A ratio with diastolic blood pressure (BP) (*top panel*) and heart rate (*bottom panel*).

Figure 3 depicts comparisons of the estimated marginal means (via linear mix model) of hemodynamic and SDPTG indices between pre- and post-match. Heart rate was higher (time effect: *p* = 0.019). Systolic and diastolic BP, and D/A ratio were lower than the pre-match value (time effects: *p* = 0.021, *p* = 0.016, and *p* = 0.023, respectively). The B/A ratio did not show a significant change over time (*p* = 0.653). However, the D/A ratio exhibited a significant effect related to the match sequence (*p* = 0.009). The D/A ratio at the third and fourth matches was lower than that at the first (Bonferroni test: *p* = 0.027 and 0.031). Rmcorr revealed that the D/A ratio exhibited significant intra-individual correlations with KES (r_rm_ = 0.200, *p* = 0.049) and RPE (r_rm_ = −0.229, *p* = 0.022) (Figure 4). Furthermore, the D/A ratio demonstrated significant intra-individual correlations with diastolic BP (r_rm_ = 0.259, *p* = 0.008) and heart rate (r_rm_ = −0.381, P< 0.001) (Figure 5). However, no significant correlation was found with systolic BP (r_rm_ = 0.155, *p* = 0.118).

## 4 Discussion

The main findings of the present study are as follows: first, during the daily repetition of soccer matches, the post-match values of the D/A ratio and BP were lower than the pre-match values, while the B/A ratio showed no significant changes. Second, the D/A ratio exhibited significant intra-individual correlations with diastolic BP and heart rate. These results suggest that, in young female football players, a brief series of matches did not increase central arterial stiffness but instead led to a repeated, temporary attenuation of arterial wave reflection, which was associated with a reduction in diastolic BP and a compensatory rise in heart rate.

In young female football players who engaged in a brief series of matches, the D/A ratio was lower 4 h after the soccer matches compared with that the night before the matches, suggesting that the attenuated arterial wave reflection lasted more than 4 h, followed by gradual recovery. Rmcorr indicated that the D/A ratio had significant intra-individual relationships with the indices of fatigue (i.e., KES and RPE). While the level of fatigue likely varied individually depending on each player’s position and the strength of the opposing team, the series of matches consistently induced repeated, temporary attenuation of arterial wave reflection, which was associated with physical exertion in each individual.

We found mild hypotension in our subjects after the matches, as depicted in Figure 3. Specifically, the D/A ratio exhibited a significant intra-individual correlation with diastolic BP but not with systolic BP. While systolic BP is influenced by several hemodynamic factors, including arterial stiffness, stroke volume, and left ventricular ejection fraction, the primary hemodynamic determinants of diastolic BP include total peripheral resistance, heart rate, arterial stiffness, and systolic BP (Tanaka et al., 2016). It is important to note that postexercise hemodynamic responses to acute endurance exercise bouts differ between men and women. Endurance-trained women typically experience postexercise hypotension due to a persistent reduction in systemic vascular resistance, whereas endurance-trained men showed a substantial drop in cardiac output with unchanged systemic vascular resistance (Senitko et al., 2002). Therefore, the prolonged reduction in peripheral vascular tone may explain the postexercise hypotension in our subjects.

Exaggerated hypotension may be unfavorable for normotensives. Orthostatic hypotension, a hemodynamic malregulation, is often observed following endurance exercise (Mundel et al., 2015) and in women (Bryarly et al., 2019), mainly due to peripheral blood pooling and insufficient offset by increases in cardiac output. It may precipitate lightheadedness, dizziness, fatigability, and presyncope (Ma et al., 2024). Fortunately, no one complained of unfavorable hypotension-related symptoms. We could speculate on the contribution of tachycardia as an underlying mechanism. The increase in heart rate lasted 4 h after the match, as did the decrease in the D/A ratio. In addition, the individual response of the D/A ratio is inversely correlated with that of heart rate, as shown in Figure 5. Thus, the increase in heart rate may partly compensate for the prolonged reduction in systemic vasodilation after the soccer match, not to drop BP substantially.

In contrast to the D/A ratio, the B/A ratio did not change significantly during the observation period. The B/A ratio seems to be associated with central arterial stiffness (Westerhof et al., 1972a; Hashimoto et al., 2002), which typically reduces 30–60 min after moderate-intensity endurance exercise (Kingwell et al., 1997). Since we did not assess SDTPG indices within a couple of hours after the soccer match, it remains uncertain whether the B/A ratio might decrease tentatively and return to the baseline level by 4 h post-match. Our previous research on well-trained male collegiate endurance runners demonstrated increased arterial stiffness and elevated aortic systolic and pulse pressures after a 7-day intense endurance training camp (Tomoto et al., 2015; 2018), reflecting heightened vascular tone after short-term, intense endurance training. However, the current study suggests a gradual decrease in peripheral vascular smooth muscle tone without significant changes in central arterial stiffness after repeated soccer matches. Taken together, these findings imply that peripheral vascular tone is more sensitive to physical exertion than central arterial stiffness in endurance-trained females.

### 4.1 Significance and perspectives

An adequate balance between fatigue (derived from training and competition load) and recovery is essential for athletes to accomplish high-level training and achieve high-level performance continuously. Therefore, systematic monitoring of athletes’ condition is essential for their performance and for preventing adverse outcomes such as under-recovery, nonfunctional overreaching, overtraining syndrome, injuries, or illnesses (Kellmann et al., 2018). The rapid and accurate collection of physiological responses to the single and repetitive exercise load may contribute to getting useful markers for athletic conditioning. Given the simplicity of the technique, SDPTG assessment holds promise as a technique suitable for routine self-monitoring of athletes’ vascular conditions. In the present study, the reduction of the D/A ratio was seen not only post-match but also at the second 2-consecutive match (vs the first match). It is likely to be associated with accumulated fatigue. Future research should further explore the response of the D/A ratio as a biomarker for under-recovery and nonfunctional overreaching due to prolonged excessive exercise training.

### 4.2 Limitations

Required running speed and distance vary in each position. Furthermore, opposing teams are different in each match. Because of its nature, we cannot control individual physiological load and fatigue. Furthermore, this study did not involve the sedentary control group. However, through repeated measures correlation analysis, we were able to clearly demonstrate the impact of accumulated fatigue on individual vascular conditions. Lastly, the results of this study are specific to young female athletes, and it remains unclear if these findings would be applicable to male athletes or different age groups.

## 5 Conclusion

A brief series of matches in young female football players induced the repeated, temporary attenuation of arterial wave reflection, which was associated with reduced diastolic BP and a compensatory increase in heart rate.

## Data Availability

The raw data supporting the conclusions of this article will be made available by the authors, without undue reservation.
